# Comparative Study of Colorimetric In Situ Hybridization and Quantitative Real-Time Polymerase Chain Reaction for Diagnosis of Infection by *Leishmania infantum* in Dogs in Formalin-Fixed and Paraffin-Embedded Skin

**DOI:** 10.3390/tropicalmed9040091

**Published:** 2024-04-22

**Authors:** Ricardo Gonçalves Silva, Matti Kiupel, Ingeborg Maria Langohr, Annabel Wise, Sandro Antonio Pereira, Natália Pedra Gonçalves, Greice Maria Silva da Conceição, Luiz Cláudio Ferreira, Monique Paiva de Campos, Luciana de Freitas Campos Miranda, Fabiano Borges Figueiredo, Raquel de Vasconcellos Carvalhaes de Oliveira, Lucas Keidel, Rodrigo Caldas Menezes

**Affiliations:** 1 Laboratory of Clinical Research on Dermatozoonoses in Domestic Animals, Evandro Chagas National Institute of Infectious Diseases, Oswaldo Cruz Foundation, Av. Brasil, 4365, Rio de Janeiro 21040-360, Brazil; gsricardo@hotmail.com (R.G.S.); sandro.pereira@ini.fiocruz.br (S.A.P.); cowboylcf321@gmail.com (L.C.F.); lucas.keidel@ini.fiocruz.br (L.K.); 2 Veterinary Diagnostic Laboratory, Michigan State University, 4125 Beaumont Road, Lansing, MI 48910, USA; kiupel@msu.edu (M.K.); wisea@msu.edu (A.W.); 3 Sanofi, Global Discovery Pathology, Translational Models Research Platform, 350 Water Street, Cambridge, MA 02141, USA; ingeborg.langohr@sanofi.com; 4 Bio-Manguinhos, Oswaldo Cruz Foundation, Av. Brasil, 4365, Rio de Janeiro 21040-360, Brazil; natalia.goncalves@bio.fiocruz.br (N.P.G.); greice.conceicao@bio.fiocruz.br (G.M.S.d.C.); 5 Carlos Chagas Institute, Oswaldo Cruz Foundation, Rua Prof. Algacyr Munhoz Mader, 3775, Curitiba 81350-010, Brazil; m.pcampos@yahoo.com.br (M.P.d.C.); fabiano.figueiredo@fiocruz.br (F.B.F.); 6 Laboratory of Clinical Research and Surveillance of Leishmaniasis, Evandro Chagas National Institute of Infectious Diseases, Oswaldo Cruz Foundation, Av. Brasil, 4365, Rio de Janeiro 21040-360, Brazil; luciana.freitas@ini.fiocruz.br; 7 Laboratory of Clinical Epidemiology, Evandro Chagas National Institute of Infectious Diseases, Oswaldo Cruz Foundation, Av. Brasil, 4365, Rio de Janeiro 21040-360, Brazil; raquel.vasconcellos@ini.fiocruz.br

**Keywords:** canine visceral leishmaniasis, colorimetric in situ hybridization, qPCR, skin, sensitivity

## Abstract

The zoonotic visceral leishmaniasis is caused by the protozoan *Leishmania infantum* and dogs are reservoirs for this parasite. For the diagnosis of *Leishmania* at the species level in dogs in formalin-fixed, paraffin-embedded skin (FFPES) samples, colorimetric in situ hybridization (CISH) and quantitative real-time polymerase chain reaction (qPCR) are options, but their sensitivities are not well established. Therefore, the aim of this study was to determine the sensitivity of these two techniques in FFPES for the diagnosis of the *L. infantum* infection in dogs using culture as the reference standard. The FFPES of 48 dogs with cutaneous infection by *L. infantum* confirmed by culture and by multilocus enzyme electrophoresis were examined by CISH and qPCR using specific probes for *L. infantum*. The sensitivities of qPCR, CISH and their combination were, respectively, 77.0%, 58.0% and 83.3%. The sensitivities of qPCR in dogs with and without clinical signs were, respectively, 74.2% and 82.4%. The sensitivities of CISH in dogs with and without clinical signs were, respectively, 61.3% and 52.9%. The CISH and qPCR showed satisfactory sensitivities for the diagnosis of *L. infantum* in the FFPES of dogs, even in dogs without clinical signs, and their combination increases the sensitivity for this diagnosis.

## 1. Introduction

The zoonotic visceral leishmaniasis (ZVL) is a disease caused by the protozoan *Leishmania* (*Leishmania*) *infantum* and is of high relevance for public health in Brazil, affecting humans, domestic and wild mammals [1]. It is a systemic, chronic, serious and potentially fatal disease, with involvement of cells from the mononuclear phagocytic system of several organs, mainly the spleen, liver, lymph nodes and bone marrow [1,2,3]. In dogs, involvement of the skin is frequent, which is therefore considered a good target for detecting the parasite and evaluating canine infectivity for the vector [4,5].

The transmission of ZVL between humans and animals in Brazil occurs mainly through the bite of sandfly vectors of the species *Lutzomyia longipalpis* and *Lutzomia cruzi*. The reservoirs of *L. infantum* in the urban environment are domestic dogs and in the wild environment foxes and marsupials [1]. This zoonosis is endemic in countries in Latin America, Asia, Africa and Europe (Mediterranean Basin) [2]. In 2022, 1684 human cases were recorded in Brazil, with 173 deaths [6].

In Brazil, one of the main control and prevention measures for ZVL recommended by the Ministry of Health is the diagnosis of the *L. infantum* infection in dogs and the euthanasia of all dogs positive for this parasite. The confirmatory diagnosis of the *L. infantum* infection in dogs is performed by laboratory techniques that include serological (ELISA and immunochromatograhic tests), parasitological (culture and cytopathology), histological (histopathology, immunohistochemistry and in situ hybridization) and PCR techniques [7,8,9,10]. All these diagnostic techniques have advantages and disadvantages and none of them are 100% sensitive and specific, especially in animals without clinical signs [9,11,12,13,14,15,16,17,18]. Therefore, it is common to combine these laboratory techniques for a more accurate diagnosis of the infectious agent [10,18].

In Brazil, in addition to *L. infantum*, dogs can be infected by *L. braziliensis* [19] and *L. amazonensis* [20], but they are not considered a reservoir for the last two *Leishmania* species [1]. Therefore, the identification of the *Leishmania* species is important for surveillance and control actions. Parasitological culture techniques followed by speciation using the multilocus enzymatic electrophoresis (MLEE) technique and PCR are the techniques routinely used for the diagnosis of *Leishmania* at the species level [21,22]. Alternatively, colorimetric in situ hybridization (CISH) using a specific probe can discriminate *L. infantum* from the other species of *Leishmania* that infect dogs in the New World [9].

Culture followed by speciation using the MLEE technique is the reference method for identifying *Leishmania* at the species level and its sensitivity is high in diagnosing ZLV in dogs (78% to 80%), when the clinical sample is properly collected and processed [4,23]. However, this technique is limited to fresh biological samples, it is time-consuming, it is susceptible to microbiological contamination, and it can be difficult to perform due to the poor adaptation of some isolates to the culture medium [7].

Polymerase chain reaction (PCR) is considered the most sensitive technique for diagnosing ZVL in dogs. Several PCR methodologies are used in the specific detection of *L. infantum* in dogs, mainly conventional PCR (cPCR), nested PCR and quantitative real-time PCR (qPCR) [24]. The qPCR technique is advantageous in relation to other PCR methodologies because it uses sample processing in a closed system and is therefore less prone to contamination. In addition, qPCR allows the quantification of the parasite load and the identification of the *Leishmania* species with a specific probe without the need for sequencing the amplicons [22,24]. The disadvantages of PCR techniques are the lack of standardization of protocols, false negative results associated with losses of DNA in the extraction protocol, presence of Taq polymerase inhibitors, non-detection of active infection, and lower sensitivity and greater difficulty in identifying *Leishmania* at the species level in FFPE tissues when compared to the use of fresh frozen tissues [9,10,18,22,24,25,26,27]. In addition, the use of some species-specific primers for *L. infantum* in cPCR can result in nonspecific amplifications of DNA [28].

The colorimetric in situ hybridization (CISH) technique uses an antigen-labeled nucleic acid probe, which specifically binds to a complementary nucleic acid sequence of the infectious agent. This binding is visualized under light microscopy via an antigen–antibody interaction and a chromogenic detection with a colored enzyme substrate [9,25]. The main advantage of CISH, when compared to PCR, is the simultaneous visualization of the amastigote forms in the tissue and associated lesions, which confirms active infection. An automated CISH using a specific probe for the *L. donovani* complex showed a good accuracy (87.2%), superior to that of histopathology and immunohistochemistry when tested in formalin-fixed and paraffin-embedded skin (FFPES) samples from dogs infected with *L. infantum* [9].

FFPES is frequently used in laboratorial routines for the diagnosis of infection by *L. infantum* in dogs because it is a good target for the detection of this parasite and does not need ideal conditions of cooling and transportation in the field, such as culturing does [9,22,29,30,31]. FFPES is often the only sample available for the diagnosis of *Leishmania* spp. In these cases, the diagnosis at species level is routinely done by PCR techniques, such as qPCR [22], and CISH using a specific probe is an alternative for detection of *L. infantum* in dogs from the New World [9]. However, comparison of the sensitivities of semi-automated CISH and qPCR techniques in detecting *L. infantum* in FFPES from dogs has not yet been performed. The evaluation of these two techniques is important for improving the detection of *L. infantum* at the species level in the FFPES of dogs and contributing to more effective control and prevention of ZVL. Therefore, the objective of this study is to evaluate the sensitivity of semi-automated CISH and qPCR techniques on FFPE intact skin samples for diagnosing the *L. infantum* infection in dogs using the parasitological culture technique as a reference standard method.

## 2. Materials and Methods

### 2.1. Samples

A retrospective study was carried out using a non-probabilistic sample of intact skin from the scapular region of 48 dogs with positive results for *L. infantum* confirmed using parasitological culture and MLEE techniques. Of these 48 dogs, 27 had clinical signs and 21 had no clinical signs.

Three-millimeter punch biopsy samples of intact skin were collected from the scapular region of dogs. The dogs came from areas endemic for ZVL in Brazil and skin samples were collected between 2008 and 2016. The cities of origin of these animals were Niterói, Rio de Janeiro, Barra Mansa and Maricá in the state of Rio de Janeiro, and Bauru in the state of São Paulo. After collection, the samples were immediately fixed in 10% buffered formalin and embedded in paraffin. Samples were then processed for CISH and qPCR in 2017. Five of the skin samples were from 2008, two from 2012, 12 from 2014, 18 from 2015, and 11 from 2016. A skin sample was also stored immediately after collection in sterile saline solution to perform parasitological culture and MLEE techniques.

Dogs with clinical signs compatible with ZVL were considered to be those that presented at least one of the following changes: thinness, cachexia, localized or general alopecia, skin lesions characterized by ulcers and/or scaling, onychogryphosis, enlargement of superficial lymph nodes, hepatomegaly, splenomegaly, pale discoloration of ocular or oral mucous membranes, keratoconjunctivitis and skeletal muscle atrophy [32,33]. The dogs were divided into two groups according to the absence or presence of clinical signs of ZVL.

### 2.2. Parasite Culture and MLEE

The skin biopsy samples immediately immersed in sterile saline solution containing antimicrobials were then seeded and cultured at 26–28 °C in Novy, MacNeal and Nicolle plus Schneider Medium Insect biphasic culture medium (Sigma–Aldrich Co., St. Louis, MO, USA) supplemented with 10% fetal bovine serum, penicillin, and streptomycin, according to the protocol described at https://dx.doi.org/10.17504/protocols.io.22tggen (accessed on 29 December 2023). The isolated promastigote forms of *Leishmania* spp. were identified as *L.* (*L.*) *infantum* by multilocus enzymatic electrophoresis (MLEE) [21].

### 2.3. Colorimetric In Situ Hybridization

Two serial sections of 5 µm thickness of the FFPES were placed on silanized slides ERV–SF PLUS^®^ (Erviegas, Indaiatuba, São Paulo, Brazil). The ZytoFast^®^ PLUS CISH implementation kit (Zytovision GmbH, Bremerhaven, Germany) was used according to the manufacturer’s recommendations. The process began by conditioning the slides in an oven at 75 °C for 20 min, followed by the deparaffinization process in xylene and rehydration in decreasing concentrations of ethanol. Afterwards, the slides were washed in running water for three minutes and then in distilled water for two minutes. After the hydration step, the slides were subjected to proteolytic treatment using pepsin, for two minutes at 37 °C, in a humid chamber. The technique continued with washing the slides in distilled water with two baths of one minute each. The cell conditioning stage took place in water bath equipment, at a temperature of 99 °C for 20 min, using the ethylenediaminetetraacetic acid (EDTA) buffer solution of the commercial kit. After cell conditioning, the slides were left at room temperature for 10 min in the same buffer and washed in distilled water (two one-minute baths), dehydrated in increasing concentrations of ethanol (70%, 80%, 90% and 100%) for one minute at each concentration and dried in an oven at 37 °C for 10 min. Then, the slides were placed in the Thermobrite^®^ hybridizer equipment (StatSpin Inc., Westwood, MA, USA), incubated with an oligonucleotide probe specific for *L. donovani complex* (5′-CCTACCCGGAGGACCAGAAAAGTT-3′) linked to digoxigenin at the 5′ end [9], at a dilution of 1:500, using the hybridization buffer solution H7782 (Sigma–Aldrich Co., St. Louis, MO, USA). This oligonucleotide probe is specific for *L. infantum* in the New World and targets a fragment of the kinetoplast minicircle DNA (kDNA) gene of the parasite [9]. For incubation with the probe, the histological sections were covered with a HybriSlip^TM^ plastic coverslip (Sigma–Aldrich Co., St. Louis, MO, USA), and sealed with Fixogum glue (Marabu GmbH & Co. KG, Bietigheim-Bissingen, Germany). In the hybridizer, denaturation was carried out at 75 °C for 5 min and, immediately afterwards, hybridization occurred at 37 °C overnight. After hybridization, the coverslips were removed and the slides were then subjected to three five-minute stringency baths in a Tris-buffered-saline solution at a concentration of 1X: the first at room temperature; the second at 55 °C; and the third at room temperature.

After the three stringency baths, the slides were incubated with anti-digoxigenin monoclonal antibody produced in rabbits for 30 min at 37 °C. Then, the sections were incubated with polymer-conjugated anti-rabbit antibody and alkaline phosphatase for 30 min at 37 °C. Subsequently, the sections were submitted to signal visualization using 5-bromo-4-chloro-3 indolyl phosphate (BCIP) and 4-nitro blue tetrazolium chloride (NBT) for 30 min at 37 °C in a dark chamber. Afterwards, the sections were counterstained with fast red nuclear dye for 5 min and mounted with Entellan^TM^ mounting medium (Merck KGaA, Darmstadt, Germany). In all tests using CISH, histological sections of tissues positively parasitized by the amastigote forms of *L. infantum* incubated with the probe acted as the positive control and similar sections exclusively incubated with the hybridization solution acted as a negative control. In addition, FFPE tissue samples of dogs negative for *Leishmania* spp. and tissue samples infected with pathogenic protozoan and fungi that represent differential diagnoses of *L. infantum* were incubated with the probe as further negative controls. These additional negative control samples consisted of two skin samples of dogs negative for *Leishmania* spp. by parasitological culture and immunohistochemistry, one spleen sample of a dog positive for *Toxoplasma gondii* by immunohistochemistry, one lymph node and one lung sample of a dog positive for *Rangelia vitalii* by histopathology, one heart sample of a dog positive for *Trypanosoma cruzi* by histopathology and immunohistochemistry, two skin samples of dogs positive for *L. braziliensis* by parasitological culture and MLEE, two skin samples of dogs positive for *Sporothrix* spp. by mycological culture and Grocott’s methenamine silver stain, and one liver sample of a dog positive for *Histoplasma capsulatum* by mycological culture and Grocott’s methenamine silver stain. In all these tissue samples used as negative controls, microorganisms were easily visible by light microscopy.

### 2.4. Reading of CISH Slides

Microscopic interpretation was performed by two observers. Observer 1 was a veterinary pathologist experienced in the histopathological, immunohistochemical, and CISH diagnosis in leishmaniasis. Observer 2 was a veterinarian who worked in quality control of immunobiologicals and had no previous experience in the histopathological, immunohistochemistry, and CISH diagnosis in leishmaniasis.

All slides were examined under an optical microscope using 40× and 100× objective lenses to search for amastigote forms of *Leishmania*. The final results of the readings were obtained by Observer 1, the more experienced of the two observers.

The samples processed by CISH were considered positive when at least one dark-blue-stained structure morphologically compatible with the parasite was observed. In addition, shape, size, texture, and location inside parasitophorous vacuoles of macrophages were considered.

### 2.5. qPCR Technique

#### 2.5.1. Extraction and Purification of DNA from FFPES

The FFPES samples were subjected to eight 5 μm thick histological sections. To avoid contamination between samples, the microtome was sanitized with 70% ethanol, and the disposable microtome blade was replaced at the beginning of sectioning of each sample. Histological sections were collected using sterile tweezers and transferred directly to 1.5 mL previously labeled microtubes (DNAse and RNAse free). Samples were deparaffinized by adding 1 mL of xylene, followed by centrifugation at 14,000 rpm for two minutes. After these steps, the supernatant was discarded, and 1 mL of 100% ethanol was added. The samples were centrifuged again at 14,000 rpm for two minutes, and the supernatant was discarded. Then, the samples were incubated at room temperature for 1 h to evaporate the ethanol. After incubation, the automatic DNA extraction process was carried out on the QIAcube^®^ equipment (Qiagen GmbH, Hilden, Germany) using the commercial QIAamp^®^ DNA FFPE Tissue kit (Qiagen GmbH, Hilden, Germany), according to the manufacturer’s recommendations.

#### 2.5.2. Quantification of DNA by Fluorimetry

After the extraction step, the DNA obtained from each sample was quantified using fluorescent dyes present in the Qubit^TM^ dsDNA HS Assay kit (Invitrogen, Carlsbad, CA, USA) and using the Qubit^®^ 2.0 Fluorometer platform (Invitrogen, Carlsbad, CA, USA), following the instructions of the manufacturer.

#### 2.5.3. Construction of the Standard Curve for the Quantification of Parasite Load

For the quantification of the parasite load, a standard curve was built with serial dilutions (10^1^ to 10^5^ parasites) of *L. infantum* DNA (MHOM/BR/1974/PP75). The DNA of 1 × 10^6^ parasites was extracted using a DNeasy^®^ Blood & Tissue kit (Qiagen GmbH, Hilden, Germany), following the manufacturer’s recommendations.

#### 2.5.4. DNA Amplification Protocol by qPCR

Triplicate DNA samples were subjected to amplification by qPCR using the 7500 Fast Real-Time PCR System™ platform (Applied Biosystems, Foster City, CA, USA) and TaqMan^TM^ MGB hydrolysis probe and primers (Applied Biosystems, Foster City, CA, USA). Samples were tested in triplicate.

The TaqMan^TM^ MGB hydrolysis probe and primers were designed to consider the conserved regions of *L. infantum* kDNA [34]. The primers LEISH-1 (5′- AACTTTTCGTGGTCCTCCGGGTAG-3′) and LEISH-2 (5′-ACCCCCAGTTTCCCGCC-3′) and the probe TaqMan^TM^ MGB (FAM-5′-AAAAATGGGTGCAGAAAT-3′-NFQ-MGB) were used. The final volume of the mixture for amplification to occur was 25 μL per well, of which 5 μL was the volume of sample used and 20 μL corresponded to the reagent solution. The reagent solution contained 12.5 μL of TaqMan^TM^ Fast Advanced Master Mix reagent (Applied Biosystems, Foster City, CA, USA), 1.5 μL of LEISH-1 and LEISH-2 primers at 900 nM, and 2.5 μL of probe at 200 nM. The reactions were carried out in a 96-well plate (Applied Biosystems, Foster City, CA, USA), which was coated with adhesive film (Applied Biosystems, Foster City, CA, USA) after adding all the components of the event. The amplification protocol adopted was as follows: one cycle of 50 °C for two minutes, one cycle of denaturation at 95 °C for 10 min, and 40 cycles of denaturation at 95 °C for 15 s and annealing/extension at 60 °C for one min. Positive and negative ultrapure water controls were included in each amplification plate and a threshold of 0.1 was established. Samples in which DNA amplification occurred after the 37th cycle were classified as undetectable. The *L. infantum* parasite load was expressed as the number of parasite genome equivalents (gEq)/ng.

#### 2.5.5. DNA Quality Test

Samples with undetectable results in the amplification were submitted to DNA quality testing using the TaqMan^TM^ Gene Expression Assay (Applied Biosystems, Foster City, CA, USA) in another singleplex qPCR. This technique consisted of a predefined pair of primers and a predefined probe for amplification of a segment of the canine gene encoding the β-actin subunit (Cf03023880_g1). A final reaction volume of 25 μL was used. The results were expressed as positive or negative and samples showing amplification were considered to be free of DNA degradation and PCR inhibitors.

### 2.6. Statistical Analysis

The data was analyzed using the free R software, version 4.2.3 [35]. The software used to obtain qPCR data was AccuSEQ™ (Applied Biosystems, Foster City, CA, USA).

The results of Observer 1 in the CISH technique were used to calculate the sensitivity. The sensitivities and respective 95% confidence intervals (95% CI) were calculated using the parasitological culture as the reference standard method.

The interobserver agreement for the CISH technique and the agreement between the CISH and qPCR techniques were estimated by the Kappa index and classified according to Landis and Koch [36]: poor (<0), slight (0–0.20), fair (0.21–0.40), moderate (0.41–0.60), substantial (0.61–0.80), almost perfect (0.81–1.00).

Comparison between positive and negative groups using CISH and qPCR techniques and groups with presence and absence of clinical signs was performed using the Fisher’s exact test.

The normality of the parasite load was rejected by the Shapiro–Wilk test at a significance level of 5%, which indicated the use of non-parametric tests for the analysis of this variable. To evaluate the difference in the distribution of *L. infantum* load obtained by qPCR according to the CISH results and clinical classification, the Wilcoxon signed-rank test was used. To describe the differences, the median and minimum and maximum values of the parasite load obtained by qPCR were described based on the CISH results and clinical classification.

A *p*-value < 0.05 indicated statistical significance.

### 2.7. Ethics Statement

The study protocol was approved by the Ethics Committee on Animal Use of the Oswaldo Cruz Foundation (CEUA/Fiocruz; Permit Numbers: LW-54/13 and L-038/08).

## 3. Results

**Figure 1:**
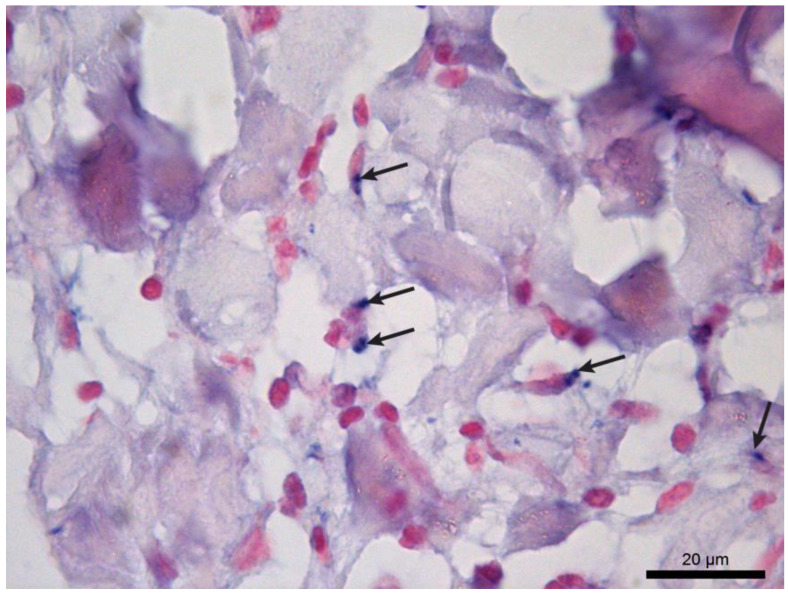
Colorimetric in situ hybridization of a dog skin sample showing amastigote forms of *L. infantum* stained in dark blue (arrows) and located in the cytoplasm of macrophages.

The sensitivity for the diagnosis of *L. infantum* established for the qPCR technique was 77.1% (95% CI 75.4–78.8%), while for the CISH technique (Figure 1) it was 58.3% (95% CI 56.3–60.3%). The sensitivity obtained with the combination of both techniques was 83.3%.

The number of positive and negative samples for each technique performed is described in Table 1. There was no cross-hybridization of the probe used in the CISH technique with any of the pathogenic microorganisms tested and in the skin samples negative for *Leishmania* spp.

Table 2 shows the sensitivity for the CISH and qPCR techniques in the samples in relation to the storage time of the paraffin blocks.

Clinical signs compatible with ZVL were present in 31 (65%) and absent in 17 (35%) of the 48 dogs evaluated. For the diagnosis of *L. infantum* infection in the skin of dogs with or without clinical signs, the sensitivity values of the CISH and qPCR techniques and the number of positive and negative samples for each technique are shown in Table 3. No statistically significant association was observed between the results of the CISH (*p*-value = 0.760) and qPCR (*p*-value = 0.723) with the clinical classification.

The Cohen Kappa Index value obtained to determine agreement between the CISH and qPCR techniques was 0.31, which indicates a fair agreement. The percentage of agreement between the techniques was 68.75% (95% CI 53.75–81.34%).

In the analysis of interobserver agreement in the CISH technique, the Cohen Kappa Index value obtained was 0.32, which indicates a fair agreement (Table 4). The percentage of interobserver agreement was 66.67% (95% CI 51.59–79.60%).

**Figure 2:**
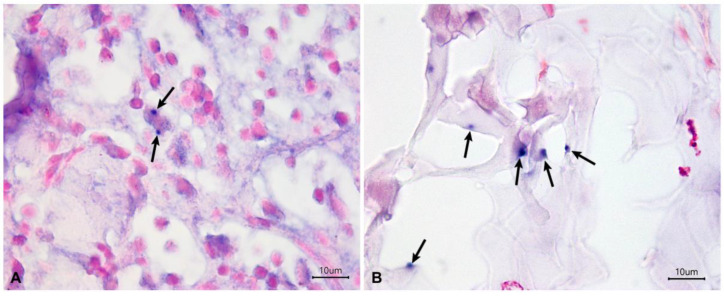
Colorimetric in situ hybridization of dog skin samples that had disagreement in the results obtained by the Observer 1 and Observer 2. (**A**) False negative case read out by the Observer 2 with two amastigote forms of *L. infantum* stained in dark blue (arrows) and located in the cytoplasm of a macrophage. (**B**) False positive case read out by the Observer 2 with dark blue artifactual staining (arrows) in the collagen fibers of dermis.

Examples of a false negative case and a false positive case read out by the Observer 2 are shown in Figure 2.

The loads of *L. infantum* in the skin determined by qPCR in dogs in the 16 cases with interobserver disagreement and in the 32 cases with interobserver agreement in the CISH technique are shown in Table 5. There was no statistically significant difference in the loads of *L. infantum* between the cases with interobserver disagreement and agreement in the CISH technique (*p*-value = 0.061).

The loads of *L. infantum* in the skin obtained by qPCR in dogs positive and negative for this parasite in the CISH are shown in Table 6. The load of *L. infantum* in the skin was significantly higher in dogs positive by CISH in comparison to dogs negative for this technique (*p*-value = 0.0035).

Of the total of 12 skin samples positive by qPCR and negative by CISH, nine had a parasite load higher than 0.079 (gEq), corresponding to the minimum value of *L. infantum* load detected in positive samples by CISH. The *L. infantum* load of these samples ranged from 0.19 to 42.71 (gEq).

The loads of *L. infantum* in the skin obtained by qPCR in dogs with and without clinical signs are shown in Table 7. There was no statistically significant association between the presence or absence of clinical signs and the load of *L. infantum* (*p*-value = 0.769).

## 4. Discussion

The qPCR technique was more sensitive than CISH in detecting *L. infantum* in samples of intact FFPE skin from dogs with and without clinical signs from an endemic area. This higher sensitivity of qPCR was expected, as it is based on the amplification of the nucleic acids of the etiological agent present in the sample, while the CISH technique aims to detect the nucleic acids of the pathogen that are already present in the sample, without amplification [25,37]. The lower frequency of positivity using the CISH technique compared to PCR was also observed by Dinhopl et al. [38], who used a generic probe for *Leishmania* spp. that targets the 5.8S ribosomal RNA gene of the parasite. These authors analyzed various FFPE tissues from 13 dogs with clinical suspicion or seropositivity for *Leishmania* spp. and showed that CISH was negative in three dogs out of a total of six positive for *Leishmania* spp. by cPCR. The low concordance between qPCR and CISH in this study was due to the differences in sensitivity obtained by these techniques.

The sensitivity of qPCR in the present study was satisfactory and was within the range of 45 to 100% reported by other authors in frozen skin samples from dogs with and without clinical signs, which were examined by cPCR and qPCR targeting the kinetoplast DNA (kDNA) of *L. infantum* [39,40,41,42]. Considering FFPES, the sensitivity of qPCR in the present study was higher than the sensitivity of 63.9% reported by Campos et al. [22], who used the same methodology as the present study, and was lower than the sensitivity of 82.8% described by Xavier et al. [43], who used cPCR and kDNA of the parasite as a target.

The lower qPCR sensitivity found by Campos et al. [22] compared to the present study was probably due to the different reference standards adopted, the clinical classification of the dogs included, and the number of histological sections collected for the qPCR. These authors adopted positive dogs in at least one of the techniques as the reference standard for calculating sensitivity: parasitological culture, immunohistochemistry (IHC), and histopathology. While in the present study the reference standard was positive dogs in parasitological culture. In addition, Campos et al. [22] did not discriminate between the clinical classification of dogs, which is a factor that influences the sensitivity of qPCR [43,44]. The number of 5 µm sections obtained from paraffin blocks for qPCR by Campos et al. [22] was five, while in our study we used eight sections. A greater number of samples may have enabled a greater amount of *L. infantum* DNA to be extracted in our study, providing a better sensitivity of qPCR compared to the results described by Campos et al. [22].

In turn, the greater sensitivity of cPCR for diagnosing *L. infantum* in the FFPES of dogs found by Xavier et al. [43] compared to our results was not expected. This is because the qPCR technique is considered to be more sensitive than cPCR [45,46]. The higher sensitivity found by Xavier et al. [43] may have been due to the different technique used, the inclusion of a convenience sample of 29 dogs (10 without clinical signs and 19 with clinical signs), lower than in the present study, as well as the use as a reference standard of dogs positive for *L. infantum* in liver, spleen or lymph node samples examined by histopathology or cytopathology. This different reference standard from Xavier et al. [43] may have led to the selection of dogs with higher parasite load in the skin, leading to a higher sensitivity than in the present study. Furthermore, Xavier et al. [43] do not mention the number of histological sections used in the cPCR, which may have influenced the sensitivity of this technique. In addition to being considered more sensitive compared to the cPCR in the diagnosis of *L. infantum* in dogs, qPCR has other advantages such as greater speed, less possibility of contamination, and information on the parasite load of the animal, which is important for monitoring the response to treatment [10,45].

The CISH technique in the present study showed sensitivity within the range of 44 to 75% expected for a histological technique (including IHC, histopathology and CISH) used to detect amastigote forms of *L. infantum* in the FFPES from naturally infected dogs [9,41,43]. Comparing the sensitivity of the histopathology in dog skin samples for diagnosing *L. infantum*, which ranges from 35.6 to 57.6% [9,41,43], CISH proved to be more sensitive in the present study, corroborating the findings of Menezes et al. [9].

Compared to the IHC for detecting *Leishmania* in the FFPES of dogs with ZVL, in the present study, CISH showed a higher sensitivity than the 44% reported by Queiroz et al. [41] and Xavier et al. [43], but lower than the 69.5% sensitivity reported by Menezes et al. [9]. Menezes et al. [9] also reported higher sensitivity for CISH compared to the present study. For CISH using a generic probe for *Leishmania* spp., the sensitivity was 70.6% and for CISH using the same probe as in the present study, the sensitivity was 74.5%. Unlike the present study, Menezes et al. [9] used an automated protocol, which increases the consistency, reliability, suitability, and productivity of the CISH technique. In addition to these properties, better tissue preservation, an improvement in the signal intensity of the detection of amastigote forms by the probe and less non-specific tissue marking (background) by the automated system may be the reason for the better sensitivity found by Menezes et al. [9] compared to the semi-automated system used in this study. In fact, in the CISH protocol of this study, damage to the skin morphology caused by the proteolytic treatment and cell conditioning steps was observed, similar to that reported by Boechat et al. [47] in epididymis samples, which may have impaired the sensitivity of this technique. Particularities of the extracellular matrix, which is composed primarily of collagen, elastin and laminin, make the histological section of the skin thin and fragile, sometimes compromising an adequate result from the CISH [48].

Although CISH in this study showed sensitivities within the range of values found for histopathology and IHC [9,41,43], the CISH has certain advantages when compared to other histological techniques. The main advantages are greater specificity, as it detects pathogen-specific nucleic acids, as well as the discrimination of *L. infantum* from other *Leishmania* spp., which is not possible with the other histological techniques. The CISH with a specific probe for *L. infantum*, which was used in this study, did not cross-hybridize with other species of New World *Leishmania*, other fungi and pathogenic protozoa in silico and in parasitized tissues and culture pellets of pathogenic FFPE microorganisms [9]. In turn, the IHC has known cross-reactions with histomorphologically similar pathogens such as *Histoplasma capsulatum* and *Sporothrix* spp. [49,50] and, in our laboratory’s experience, with *Trypanosoma cruzi*. Additionally, for IHC, there is a lack of commercial anti-*Leishmania* spp. antibodies available [9]. Therefore, the antibodies generally used are rabbit anti-*Leishmania* polyclonal serum produced in house or dog anti-*Leishmania* hyperimmune serum obtained from naturally infected dogs [43,50]. These in-house produced antibodies hinder the standardization of the technique between laboratories, and increase the risk of cross-reaction with other pathogens.

The sensitivity of CISH was related to the parasite load, with positive cases having a higher parasite load than negative cases using this technique. This technique was unable to detect amastigote forms of *Leishmania* in the skin samples with a parasite load of less than 0.079 (gEq) of *Leishmania* DNA, detected in 37 quantification cycles by qPCR. The non-detection of amastigote forms of *L. infantum* by the CISH in nine cases with a parasite load of more than 0.079 (gEq) may have been due to tissue destruction caused by the enzymatic treatment and heat treatment stages. This tissue damage may have prevented the visualization of amastigote forms present in the histological section, corroborating the findings of Boechat et al. [47].

The fair agreement between the readings of the slides processed using CISH by the two observers in this study points to a limitation of the technique, whose sensitivity results depend on the experience of the observer. According to the results of this study, the disagreement between the two observers may have been influenced by the lower parasite load detected by qPCR in these cases compared to the higher load in the cases with interobserver agreement, although no statistically significant difference was found. These results differ from the findings of Ferreira et al. [50], who reported substantial or almost perfect agreement between three readers with different degrees of experience for the CISH using a generic probe for *Leishmania* spp. in the diagnosis of American tegumentary leishmaniasis in human skin samples. The reason for these different results may have been the degree of variation between the experiences of the observers in the two studies. Additionally, in the study by Ferreira et al. [50], there may have been a better performance of the generic probe for *Leishmania* spp. compared to the specific probe for *L. infantum* used in the present study, characterized by a marking of the amastigote forms with a more intense signal and more easily visible to the observer under light microscopy. Therefore, the training of observers would be a strongly recommended measure to minimize discrepancies between the results of the reading of the slides processed by CISH in the present study.

Of the parasitological culture-positive cases, 17% were not detected either by CISH or by qPCR, demonstrating sensitivity flaws of these techniques. This deficiency in the detection of positive cases by both CISH and qPCR may be related to the process of fixation in 10% formalin, embedding in paraffin and storage time of the paraffin blocks. Reinforcing this hypothesis, Campos et al. [22] found a sensitivity of 63.6% for the qPCR technique in FFPES and 100% in frozen skin samples for detecting *L. infantum* DNA in dogs. These authors also found that the concentration of extracted DNA was significantly lower in qPCR in FFPES than in frozen skin samples, which interfered with the sensitivity of this technique. According to Maes et al. [25], fixation in 10% formalin leads to the formation of protein cross-links to DNA, causing its chemical modification. In this process, there is also the formation of cross-links between proteins that lead to DNA entrapment and fragmentation. Embedding samples in poor quality paraffin and at temperatures higher than 60 °C can also cause damage to nucleic acids. In addition, storing paraffin blocks for more than 4 years leads to DNA degradation of the samples, which can impair the sensitivity of CISH and qPCR [25,50,51]. The results of higher sensitivities of CISH and qPCR in the younger blocks (1 to 2 years of storage) compared with the older blocks (3 to 9 years of storage) in the present study reinforce this hypothesis. Despite the negative interference with the sensitivity of the CISH and qPCR, FFPE samples have important advantages over fresh or frozen samples. Among these advantages is the possibility of storing them without refrigeration, which facilitates their transportation and conservation in places with little structure [52]. In addition, due to its ease of collection and storage, low cost, and the possibility of retrospective studies, the FFPE tissue sample is often the only one sent for diagnosis of *L. infantum* in dogs in our experience as a reference center for the diagnosis of leishmaniasis in animals.

Although CISH and qPCR showed different sensitivity values in detecting *L. infantum* in dogs without clinical signs compared to sick dogs, there was no statistically significant difference between these results. Xavier et al. [43] also found no significant difference between the sensitivity of the evaluated techniques and clinical signs, although they also found a greater sensitivity of cPCR in the FFPES of dogs without clinical signs, unlike the histological techniques. Queiroz et al. [41] did not observe a difference in sensitivity of the cPCR technique in skin samples between dogs with and without clinical signs, but they found greater sensitivity in dogs with clinical signs using histological techniques, although these results were not statistically significant. The similarity of the results regarding the frequency of positivity using the qPCR technique among dogs with clinical signs was also observed by Carvalho et al. [44]. However, Furtado et al. [31] found in FFPES that the frequency of detection of amastigote forms by the CISH, using the same specific probe as in the present study in an automated protocol, was significantly higher in dogs with clinical signs (81%) compared to those without signs (47%). Taken together, the results of the present study and those of the authors mentioned above demonstrate that the PCR and CISH can identify *L. infantum* in skin samples from dogs without clinical signs. These results demonstrate the usefulness of carrying out these diagnostic techniques as disease control measures, since dogs without clinical signs can be a source of infection for the vector and are more difficult to diagnose using serological methods [44].

The CISH and qPCR are laboratorial techniques that need expensive equipment, reagents, and infrastructure. Furthermore, their sample processing is complex. Therefore, these techniques are more suitable for reference centers, while not really applicable to rural health posts. Nonetheless, the satisfactory sensitivities obtained by both techniques in this study on FFPES show that they are useful techniques for diagnosing the *L. infantum* species in situations in which parasitological culture is unfeasible and the only biological sample available is FFPE tissue. Parasitological culture is restricted to a few reference centers in Brazil, and it is common for samples to be lost due to microbiological contamination, especially those collected in the field and in regions far from the test site [9]. In this context, of the two techniques evaluated in this study, qPCR proved to be the most suitable as an alternative to parasitological culture, due to its greater sensitivity. CISH is an alternative in cases in which qPCR is not available or when the results are inconclusive due to the presence of reaction inhibitors in the sample, such as hemoglobin [25]. However, the combination of the two techniques is best recommended due to the increased sensitivity in detecting *L. infantum* in the skin of dogs. It includes the ability of CISH to identify active infection by allowing visualization of the amastigote forms of *Leishmania* in the tissues and their correlation with the tissue lesions found, which is not possible with qPCR [9,10,25].

## 5. Conclusions

The CISH and qPCR each showed satisfactory sensitivities for the diagnosis of *L. infantum* in the FFPES of dogs, even in dogs without clinical signs, and their combination increases the sensitivity for this diagnosis.

## Figures and Tables

**Table 1:** Positive and negative cases for amastigote forms of *L. infantum* by CISH and qPCR techniques in FFPE skin samples from dogs with *L. infantum* infection confirmed by parasitological culture.

CISH	qPCR		Total
	Positive	Negative	
Positive	25	3	28
Negative	12	8	20
Total	37	11	48

CISH: Colorimetric in situ hybridization; qPCR: quantitative real-time PCR.

**Table 2:** Sensitivity for the detection of *L. infantum* found by CISH and qPCR according to the time of storage of 48 paraffin blocks from dogs naturally infected.

Time of Storage (Years)	N	Sensitivity (C.I. 95%)	
		CISH	qPCR
3–9	19	42.1% (20.2–66.5%), n = 8	63.2% (38.4–83.7%), n = 12
1–2	29	69.0% (49.2–84.7%), n = 20	86.2% (68.3–96.1%), n = 25

CISH: Colorimetric in situ hybridization; N: total number of samples; n: number of positive samples; qPCR: quantitative real-time PCR; C.I.: confidence interval.

**Table 3:** Sensitivity of CISH and qPCR techniques for detecting *L. infantum* and the number of positive and negative samples for each technique according to the clinical classification of the dogs.

Technique	Dogs with Clinical Signs (n = 31)			Dogs without Clinical Signs (n = 17)		
	Sensitivity (C.I. 95%)	Positive	Negative	Sensitivity (C.I. 95%)	Positive	Negative
CISH	61.3% (42.2–78.2%)	19	12	52.9% (27.8–77.0%)	9	8
qPCR	74.2% (55.4–88.1%)	23	8	82.4% (56.6–96.2%)	14	3

CISH: Colorimetric in situ hybridization; C.I.: confidence interval; n: number of dogs.

**Table 4:** Results obtained by the two observers for the CISH technique on FFPES samples from dogs with *L. infantum* infection confirmed by parasitological culture.

CISH 1	CISH 2				Total	
	Positive		Negative			
Positive	19	39.58%	9	18.75%	28	58.33%
Negative	7	14.58%	13	27.08%	20	41.67%
Total	26	54.17%	22	45.83%	48	100.00%

CISH 1: Observer 1 of the colorimetric in situ hybridization technique; CISH 2: Observer 2 of the colorimetric in situ hybridization technique.

**Table 5:** Loads of *L. infantum* determined by qPCR and expressed in parasite genomic equivalents (gEq) in the skin of the cases with interobserver disagreement and agreement in the CISH technique.

CISH	qPCR			
	Dogs	Load of *L. infantum* (gEq)		
	N	Median	Minimum	Maximum
Interobserver agreement	32	56.8	0.0023	371,632.7
Interobserver disagreement	16	5.4	0.0792	41.6100

CISH: Colorimetric in situ hybridization; qPCR: quantitative real-time PCR; N: number of samples.

**Table 6:** Loads of *L. infantum* determined by qPCR and expressed in parasite genomic equivalents (gEq) in skin samples positive and negative for *L. infantum* by the CISH.

CISH	qPCR			
	Positive	Load of *L. infantum* (gEq)		
	N	Median	Minimum	Maximum
Positive	25	56.8	0.079225	371,632.7
Negative	12	4.6	0.002287	42.71348

CISH: Colorimetric in situ hybridization; qPCR: quantitative real-time PCR; N: number of samples.

**Table 7:** Loads of *L. infantum* in the skin of dogs determined by qPCR and expressed in parasite genomic equivalents (gEq) according to the presence or absence of clinical signs.

Clinical Signs	Load of *L. infantum* (gEq)			
	Mean	Minimum	Maximum	Median
Present (N = 23)	16,982.8	0.0000	371,632.7	12.8
Absent (N = 14)	362.4	0.0063	2708.3	14.6

## Data Availability

The data presented in this study are available upon request.
